# Comparison of regression models for estimation of isometric wrist joint torques using surface electromyography

**DOI:** 10.1186/1743-0003-8-56

**Published:** 2011-09-26

**Authors:** Amirreza Ziai, Carlo Menon

**Affiliations:** 1 MENRVA Research Group, School of Engineering Science, Faculty of Applied Science, Simon Fraser University, 8888 University Drive, Burnaby, BC, V5A 1S6, Canada

## Abstract

**Background:**

Several regression models have been proposed for estimation of isometric joint torque using surface electromyography (SEMG) signals. Common issues related to torque estimation models are degradation of model accuracy with passage of time, electrode displacement, and alteration of limb posture. This work compares the performance of the most commonly used regression models under these circumstances, in order to assist researchers with identifying the most appropriate model for a specific biomedical application.

**Methods:**

Eleven healthy volunteers participated in this study. A custom-built rig, equipped with a torque sensor, was used to measure isometric torque as each volunteer flexed and extended his wrist. SEMG signals from eight forearm muscles, in addition to wrist joint torque data were gathered during the experiment. Additional data were gathered one hour and twenty-four hours following the completion of the first data gathering session, for the purpose of evaluating the effects of passage of time and electrode displacement on accuracy of models. Acquired SEMG signals were filtered, rectified, normalized and then fed to models for training.

**Results:**

It was shown that mean adjusted coefficient of determination $\left({\text{R}}_{\text{a}}^{2}\right)$ values decrease between 20%-35% for different models after one hour while altering arm posture decreased mean ${\text{R}}_{\text{a}}^{2}$ values between 64% to 74% for different models.

**Conclusions:**

Model estimation accuracy drops significantly with passage of time, electrode displacement, and alteration of limb posture. Therefore model retraining is crucial for preserving estimation accuracy. Data resampling can significantly reduce model training time without losing estimation accuracy. Among the models compared, ordinary least squares linear regression model (OLS) was shown to have high isometric torque estimation accuracy combined with very short training times.

## Background

SEMG is a well-established technique to non-invasively record the electrical activity produced by muscles. Signals recorded at the surface of the skin are picked up from all the active motor units in the vicinity of the electrode [1]. Due to the convenience of signal acquisition from the surface of the skin, SEMG signals have been used for controlling prosthetics and assistive devices [2-7], speech recognition systems [8], and also as a diagnostic tool for neuromuscular diseases [9].

However, analysis of SEMG signals is complicated due to nonlinear behaviour of muscles [10], as well as several other factors. First, cross talk between the adjacent muscles complicates recording signals from a muscle in isolation [11]. Second, signal behaviour is very sensitive to the position of electrodes [12]. Moreover, even with a fixed electrode position, altering limb positions have been shown to have substantial impact on SEMG signals [13]. Other issues, such as inherent noise in signal acquisition equipment, ambient noise, skin temperature, and motion artefact can potentially deteriorate signal quality [14,15].

The aforementioned issues necessitate utilization of signal processing and statistical modeling for estimation of muscle forces and joint torques based on SEMG signals. Classification [16] and regression techniques [17,18], as well as physiological models [19,20], have been considered by the research community extensively. Machine learning classification methods in aggregate have proven to be viable methods for classifying limb postures [21] and joint torque levels [22]. Park et al. [23] compared the performance of a Hill-based muscle model, linear regression and artificial neural networks for estimation of thumb-tip forces under four different configurations. In another study, performance of a Hill-based physiological muscle model was compared to a neural network for estimation of forearm flexion and extension joint torques [24]. Both groups showed that neural network predictions are superior to Hill-based predictions, but neural network predictions are task specific and require ample training before usage. Castellini et al. [22] and Yang et al. [25], in two distinct studies, estimated grasping forces using artificial neural networks (ANN), support vectors machines (SVM) and locally weighted projection regression (LWPR). Yang concluded that SVM outperforms ANN and LWPR.

This study was intended to compare performance of commonly utilized regression models for isometric torque estimation and identify their merits and shortcomings under circumstances where the accuracy of predictive models has been reported to be compromised. Wrist joint was chosen as its functionality is frequently impaired due to aging [26] or stroke [7], and robots (controlled by SEMG signals) are developed to train and assist affected patients [2,3]. Performance of five different models for estimation of isometric wrist flexion and extension torques are compared: a physiological based model (PBM), an ordinary least squares linear regression model (OLS), a regularized least squares linear regression model (RLS), and three machine learning techniques, namely SVM, ANN, and LWPR.

### Physiological Based Model

Physiological based model (PBM) used in this study is a neuromusculoskeletal model used for estimation of joint torques from SEMG signals. Rectified and smoothed SEMG signals have been reported to result in poor estimations of muscle forces [27,28]. This is mainly due to (a) existence of a delay between SEMG and muscle tension onset (electromechanical delay) and (b) the fact that SEMG signals have a shorter duration than resulting forces. It has been shown that muscle twitch response can be modeled well by using a critically damped linear second order differential equation [29]. However since SEMG signals are generally acquired at discrete time intervals, it is appropriate to use a discretized form. Using backward differences, the differential equation takes the form of a discrete recursive filter [30]:

(1) $$\begin{array}{c} {\text{u}}_{\text{j}}\left(\text{t}\right)=\\ \alpha {\text{e}}_{\text{j}}\left(\text{t}-\text{d}\right)-\beta_{1}{\text{u}}_{\text{j}}\left(\text{t}-1\right)-\beta_{2}{\text{u}}_{\text{j}}\left(\text{t}-2\right) \end{array}$$

where e_j _is the processed SEMG signal of muscle j at time t, d is the electromechanical delay, α is the gain coefficient, u_j_(t) is the post-processed SEMG signal at time t, and β_1 _and β_2 _the recursive coefficients for muscle j.

Electromechanical delay was allowed to vary between 10 and 100 ms as that is the range for skeletal muscles [31]. The recursive filter maps SEMG values e_j_(t) for muscle j into post-processed values u_j_(t). Stability of this equation is ensured by satisfying the following constraints [32]:

(2) $$\begin{array}{c} \beta_{1}={\text{C}}_{1}+{\text{C}}_{2}\\ \beta_{2}={\text{C}}_{1}\times {\text{C}}_{2}\\ |{\text{C}}_{1}|<1\\ |{\text{C}}_{2}|<1 \end{array}$$

Unstable filters will cause u_j_(t) values to oscillate or even go to infinity. To ensure stability of this filter and restrict the maximum neural activation values to one, another constraint is imposed:

(3) $$\alpha -\beta_{1}-\beta_{2}=1$$

Neural activation values are conventionally restricted to values between zero and one, where zero implies no activation and one translates to full voluntary activation of the muscle.

Although isometric forces produced by certain muscles exhibit linear relationship with activation, nonlinear relationships are observed for other muscles. Nonlinear relationships are mostly witnessed for forces of up to 30% of the maximum isometric force [33]. These nonlinear relationships can be associated with exponential increases in firing rate of motor units as muscle forces increase [34]:

(4) $${\text{a}}_{\text{j}}\left(\text{t}\right)=\frac{{\text{e}}^{\text{A}{\text{u}}_{\text{j}}\left(\text{t}\right)}-1}{{\text{e}}^{\text{A}}-1}$$

where A is called the non-linear shape factor. A = -3 corresponds to highly exponential behaviour of the muscle and A = 0 corresponds to a linear one.

Once nonlinearities are explicitly taken into account, isometric forces generated by each muscle at neutral joint position at time t are computed using [35]:

(5) $${\text{F}}_{\text{j}}\left(\text{t}\right)={\text{F}}_{\max,\text{j}}\times {\text{a}}_{\text{j}}\left(\text{t}\right)$$

where F_max,j _is the maximum voluntary force produced by muscle j.

Isometric joint torque is computed by multiplying isometric force of each muscle by its moment arm:

(6) $$\tau_{\text{j}}\left(\text{t}\right)={\text{F}}_{\text{j}}\left(\text{t}\right)\times \text{M}{\text{A}}_{\text{j}}$$

where MA_j _is moment arm at neutral wrist position for muscle j and τ_j_(t) is the torque generated by muscle j at time t. Moment arms for flexors and extensors were assigned positive and negative signs respectively to maintain consistency with measured values.

As not all forearm muscles were accessible by surface electrodes, each SEMG channel was assumed to represent intermediate and deep muscles in its proximity in addition to the surface muscle it was recording from. Torque values from each channel were then scaled using mean physiological cross-section area (PCSA) values tabulated by Jacobson et al. and Lieber et al. [36-38]. Joint torque estimation values have been shown not to be highly sensitive to muscle PCSA values and therefore these values were fixed and not a part of model calibration [39]. The isometric torque at the wrist joint was computed by adding individual scaled torque values:

(7) $$\tau_{\text{e}}\left(\text{t}\right)=\sum_{\text{j}=1}^{\text{M}}\frac{\Sigma \text{PCS}{\text{A}}_{\text{j}}}{\text{PCS}{\text{A}}_{\text{j}}}\times \tau_{\text{j}}\left(\text{t}\right)$$

where M is the number of muscles used in the model, and ΣPCSA_j _is the summation of PCSA of the muscle represents by muscle j and PCSA of muscle j itself.

**Figure 1:**
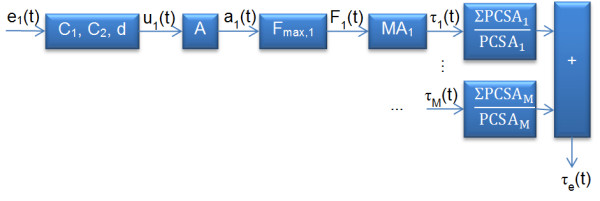
**Steps and parameters involved in the PBM**.

EDC, ECU, ECRB, PL, and FDS represented extensor digiti minimi (EDM), extensor indicis proprius (EIP), extensor pollicis longus (EPL), flexor pollicis longus (FPL), and flexor digitorum profundus (FDP) respectively due to their anatomical proximity [40]. Abductor pollicis longus (APL) and extensor pollicis brevis (EPB) contribute negligibly to wrist torque generation due to their small moment arms and were not considered in the model [41]. Steps and parameters involved in the PBM are summarized in Figure 1.

Models were calibrated to each volunteer by tuning model parameters. Yamaguchi [42] has summarized maximum isometric forces reported by different investigators. We used means as initial values and constrained F_max _to one standard deviation of the reported values. Initial values for moment arms were fixed to the mean values in [43], and constrained to one standard deviation of the values reported in the same reference. Since these parameters are constrained within their physiologically acceptable values, calibrated models can potentially provide physiological insight [24]. Activation parameters A, C_1_, C_2_, and d were assumed to be constant for all muscles a model with too many parameters loses its predictive power due to overfitting [44]. Parameters x = {A, C_1_, C_2_, d, F_max,1_, ..., F_max,M_, MA_1_, MA_2_, ..., MA_M_} were tuned by optimizing the following objective function while constraining parameters to values mentioned beforehand:

(8) $$\min_{\text{X}}{\left(\tau_{\text{e}}\left(\text{t}\right)-\tau_{\text{m}}\left(\text{t}\right)\right)}^{2}$$

Models were optimized by Genetic Algorithms (GA) using MATLAB Global Optimization Toolbox (details of GA implementation are available in [45]). GA has previously been used for tuning muscle models [20]. Default MATLAB GA parameters were used and models were tuned in less than 100 generations (MATLAB default value for the number of optimization iterations) [46].

### Ordinary Least Squares Linear Regression Model

torques using processed SEMG signals [23]. Linear regression is presented as:

(9) $${\left[\tau_{\text{m}}\right]}_{N\times 1}={\left[\text{SEMG}\right]}_{N\times M}{\left[\beta \right]}_{M\times 1}+{\left[\varepsilon \right]}_{N\times 1}$$

where N is the number of samples considered (observations), M is the number of muscles, τ_m _is a vector of measured torque values, SEMG is a matrix of processed SEMG signals, β is a vector of regression coefficients to be estimated, and ε is a vector of independent random variables.

Ordinary least squares (OLS) method is most widely used for estimation of regression coefficients [47]. Estimated vector of regression coefficients using least squares method $\left(\hat{\beta }\right)$ is computed using:

(10) $$\hat{\beta }={\left[{\left[\text{SEMG}\right]}^{\text{T}}\left[\text{SEMG}\right]\right]}^{-1}{\left[\text{SEMG}\right]}^{\text{T}}\left[\tau_{\text{m}}\right]$$

Once the model is fitted, SEMG values can be used for estimation of torque values (τ_e_) as shown:

(11) $${\left[\tau_{\text{e}}\right]}_{N\times 1}={\left[\text{SEMG}\right]}_{N\times M}{\hat{\beta }}_{M\times 1}$$

### Regularized Least Squares Linear Regression Model

The ℓ_1_-regularized least squares (RLS) method for estimation of regression coefficients is known to overcome some of the common issues associated with the ordinary least squares method [48]. Estimated vector of regression coefficients using ℓ_1_-regularized least squares method $\left(\hat{\beta }\right)$ is computed through the following optimization:

(12) $$\begin{array}{c} minimize\overset{M}{\underset{i=1}{\sum }}\lambda |{\hat{\beta }}_{i}|\\ +\sum_{i=1}^{N}{\left(\begin{array}{c} {\left[\text{SEMG}\right]}_{N\times M}{\left[\hat{\beta }\right]}_{M\times 1}\\ +{\left[\varepsilon \right]}_{N\times 1}-{\left[\tau_{\text{m}}\right]}_{N\times 1} \end{array}\right)}^{2} \end{array}$$

where *λ *≥ 0 is the regularization parameter which is usually set equal to 0.01 [49,50].

We used the Matlab implementation of the ℓ_1_-regularized least squares method [51].

### Support Vector Machines

Support vectors machines (SVM) are machine learning methods used for classification and regression. Support vector regression (SVR) maps input data using a non-linear mapping to a higher-dimensional feature space where linear regression can be applied. Unlike neural networks, SVR does not suffer from the local minima problem since model parameter estimation involves solving a convex optimization problem [52].

We used epsilon support vector regression (ε-SVR) available in the LibSVM tool for Matlab [53]. Details of ε-SVR problem formulation are available in [54]. ε-SVR has previously been utilized for estimation of grasp forces [22,25]. The Gaussian kernel was used as it enables nonlinear mapping of samples and has a low number of hyperparameters, which reduces complexity of model selection [55]. Eight-fold cross-validation to generalize error values and grid-search for finding the optimal values of hyperparameters C, γ and ε were carried out for each model.

### Artificial Neural Networks

**Figure 2:**
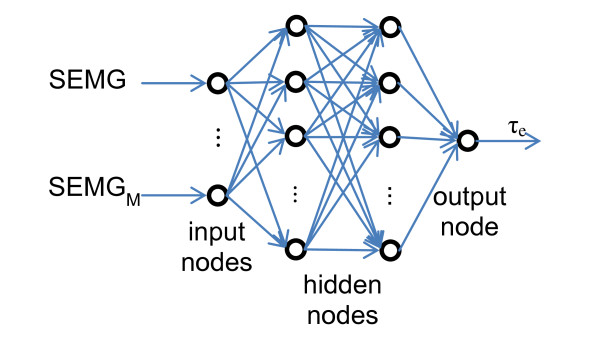
**ANN structure**.

Artificial neural networks (ANN) have been used for SEMG classification and regression extensively [22,25,56,57]. Three layer neural networks have been shown to be adequate for modeling problems of any degree of complexity [58]. We used feed-forward back propagation network with one input layer, two hidden layers, and one output layer [59]. We also used BFGS quasi-Newton training that is much faster and more robust than simple gradient descent [60]. Network structure is depicted in Figure 2, where M is the number of processed SEMG channels used as inputs to the ANN and τ_e _is the estimated torque value.

ANN models were trained using Matlab Neural Network Toolbox. Hyperbolic tangent sigmoid activation functions were used to capture the nonlinearities of SEMG signals. For each model, the training phase was repeated ten times and the best model was picked out of those repetitions in order to overcome the local minima problem [52]. We also used early stopping and regularization in order to improve generalization and reduce the likelihood of overfitting [61].

### Locally Weighted Projection Regression

Locally Weighted Projection Regression (LWPR) is a nonlinear regression method for high-dimensional spaces with redundant and irrelevant input dimensions [62]. LWPR employs nonparametric regression with locally linear models based on the assumption that high dimensional data sets have locally low dimensional distributions. However piecewise linear modeling utilized in this method is computationally expensive with high dimensional data.

We used Radial Basis Function (RBF) kernel and meta-learning and then performed an eight-fold cross validation to avoid overfitting. Finally we used grid search to find the initial values of the distance metric for receptive fields, as it is customary in the literature [22,25]. Models were trained using a Matlab version of LWPR [63].

## Methods

**Figure 3:**
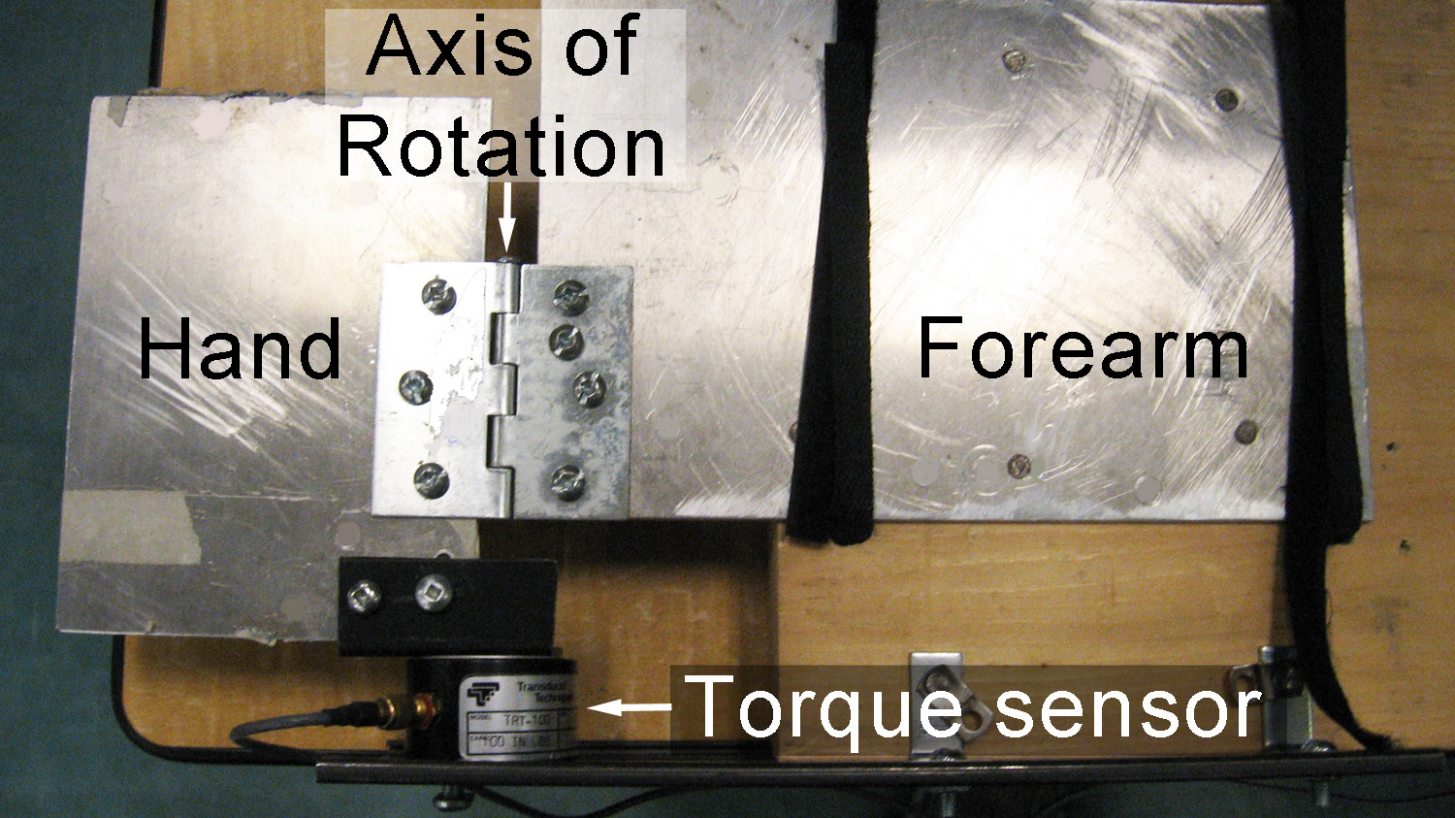
**Custom-built rig equipped with a torque sensor**.

A custom-built rig was designed to allow for measurement of isometric torques exerted about the wrist joint. Volunteers placed their palm on a plate and Velcro straps were used to secure their hand and forearm to the plate. The plate hinged about the axis of rotation shown in Figure 3.

A Transducer Techniques TRX-100 torque sensor, with an axis of rotation corresponding to that of the volunteer's wrist joint, was used to measure torques applied about the wrist axis of rotation. Volunteer's forearm was secured to the rig using two Velcro straps. This design allowed restriction of arm movements. Volunteer placed their elbow on the rig and assumed an upright position.

### Protocol

**Figure 4:**
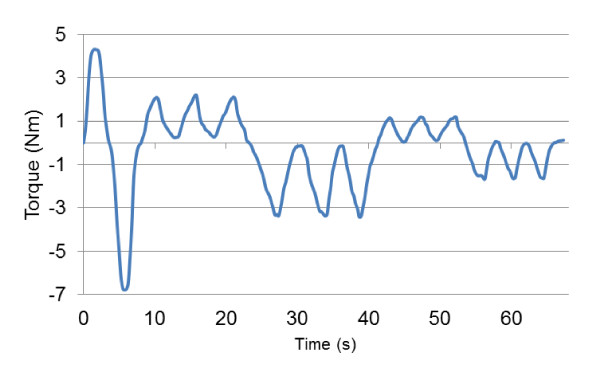
**Sample torque signal**.

Eleven healthy volunteers (eight males, three females, age 25 ± 4, mass 74 ± 12 kg, height 176 ± 7 cm), who signed an informed consent form (project approved by the Office of Research Ethics, Simon Fraser University; Reference # 2009s0304), participated in this study. Each volunteer was asked to flex and then extend her/his right wrist with maximum voluntary contraction (MVC). Once the MVC values for both flexion and extension were determined, the volunteer was asked to gradually flex her/his wrist to 50% of MVC. Once the 50% was reached the volunteer gradually decreased the exerted torque to zero. This procedure was repeated three times for flexion and then for extension. Finally the volunteer was asked to flex and extend her/his wrist to 25% of MVC three times. Figure 4 shows a sample of torque signals gathered. Positive values on the scale are for flexion and negative values are for extension.

**Figure 5:**
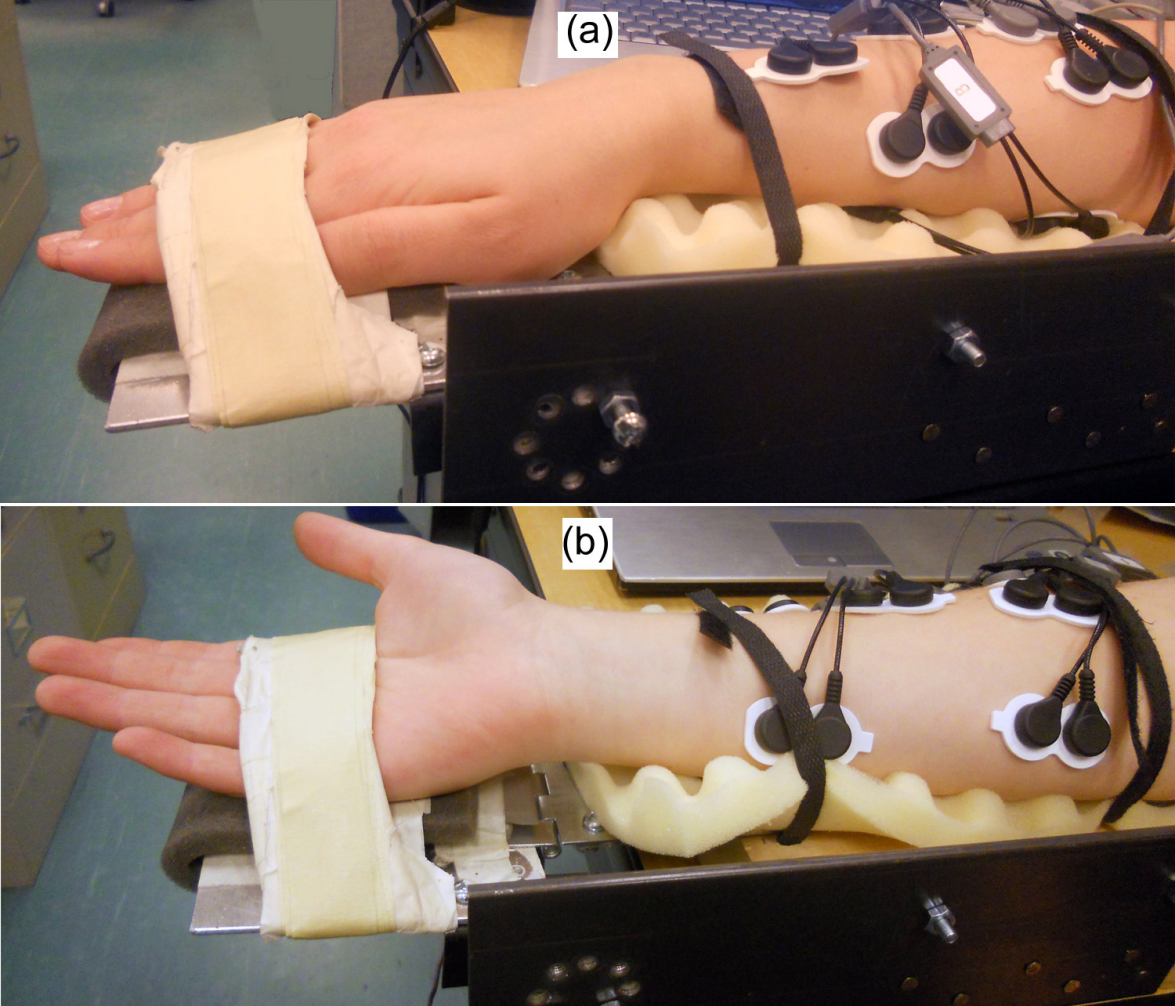
**Volunteer's forearm on the testing rig**. (a) Forearm pronated. (b) Forearm supinated.

Following the completion of this protocol, volunteers were asked to supinate their forearm, and follow the same protocol as before. Figure 5 shows forearm in pronated and supinated positions.

Completion of protocols in both pronated and supinated forearm positions was called a session. Table 1 summarizes actions in protocols.

**Table 1:** Actions and repetitions for protocols.

Repetition	Action
1	Wrist flexion with maximum torque

1	Wrist extension with maximum torque

3	Gradual wrist flexion until 50% MVC and gradual decrease to zero

3	Gradual wrist extension until 50% MVC and gradual decrease to zero

3	Gradual wrist flexion until 25% MVC and gradual decrease to zero

3	Gradual wrist extension until 25% MVC and gradual decrease to zero

In order to capture the effects of passage of time on model accuracy, volunteers were asked to repeat the same session after one hour. This session was named session two. Electrodes were not detached in between the two sessions. After completion of session two, electrodes were removed from the volunteer's skin. The volunteer was asked to repeat another session in twenty four hours following session two while attaching new electrodes. This was intended to capture the effects of electrode displacement and further time passage.

Each volunteer was asked to supinate her/his forearm and exert isometric torques on the rig following the same protocol used before after completion of session 1. This was intended to study the effects of arm posture on model accuracy.

### SEMG Acquisition

A commercial SEMG acquisition system (Noraxon Myosystem 1400L) was used to acquire signals from eight SEMG channels. Each channel was connected to a Noraxon AgCl gel dual electrode that picked up signals from the muscles tabulated in Table 2.

**Table 2:** Muscles monitored using SEMG.

Channel	Muscle	Action
1	Extensor Carpi Radialis Longus (ECRL)	Wrist extension Radial deviation

2	Extensor Digitorum Communis (EDC)	Wrist extension Four fingers extension

3	Extensor Carpi Ulnaris (ECU)	Wrist extension Ulnar deviation

4	Extensor Carpi Radialis Brevis (ECRB)	Wrist extension Wrist abductor

5	Flexor Carpi Radialis (FCR)	Wrist flexion Radial deviation

6	Palmaris Longus (PL)	Wrist flexion

7	Flexor Digitorum Superficialis (FDS)	Wrist flexion

8	Flexor Carpi Ulnaris (FCU)	Wrist flexion Ulnar deviation

**Figure 6:**
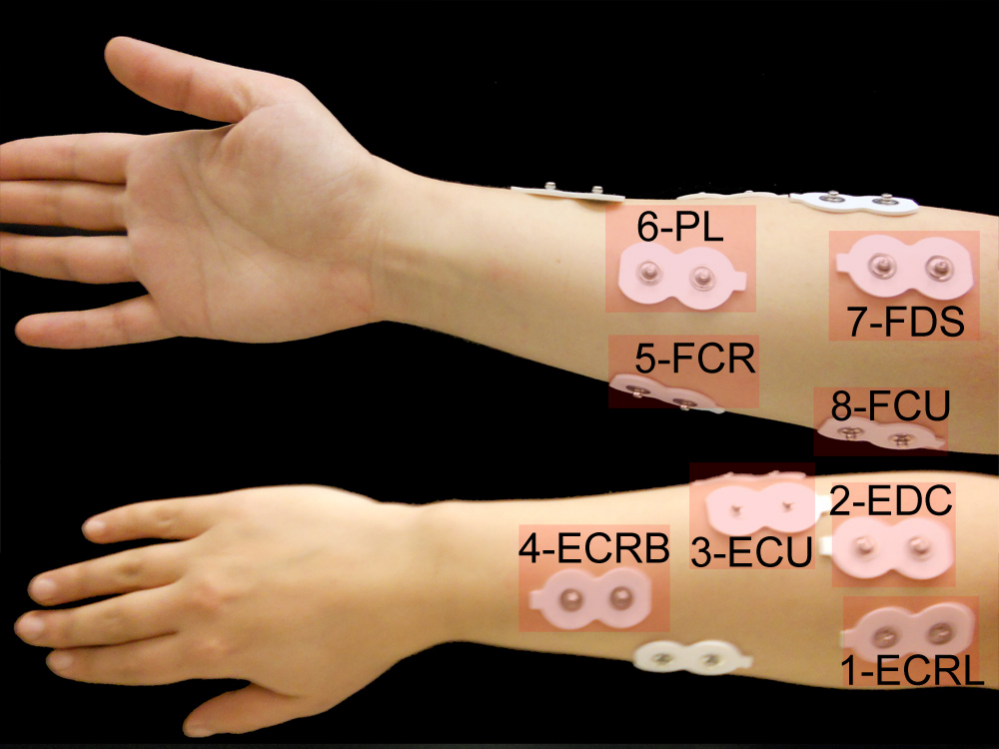
**Electrode positions**.

It has been reported that the extrinsic muscles of the forearm have large torque generating contributions in isometric flexion and extension [64]. Therefore we considered three superficial secondary forearm muscles as well as the primary forearm muscles accessible via SEMG. The skin preparation procedure outlined in surface electromyography for the non-invasive assessment of muscles project (SENIAM) was followed to maximize SEMG signal quality [65]. Figure 6 shows the position of electrodes attached to a volunteer's forearm.

SEMG signals were acquired at 1 kHz using a National Instruments (NI-USB-6289) data acquisition card. An application was developed using LabVIEW software that stored data on a computer and provided visual feedback for volunteers. Visual feedback consisted of a bar chart that visualized the magnitude of exerted torques, which aided volunteers to follow the protocol more accurately.

### Signal Processing

**Figure 7:**
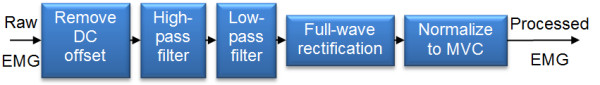
**SEMG signal processing scheme**.

Initially DC offset values of SEMG signals were removed. Signals were subsequently high-pass filtered using a zero-lag Butterworth fourth order filter (30 Hz cut-off frequency), in order to remove motion artefact. Signals were then low-pass filtered using a zero-lag Butterworth fourth order filter (6 Hz cut-off frequency), full-wave rectified and normalized to the maximum SEMG value for each channel. Figure 7 shows the signal processing scheme.

**Figure 8:**
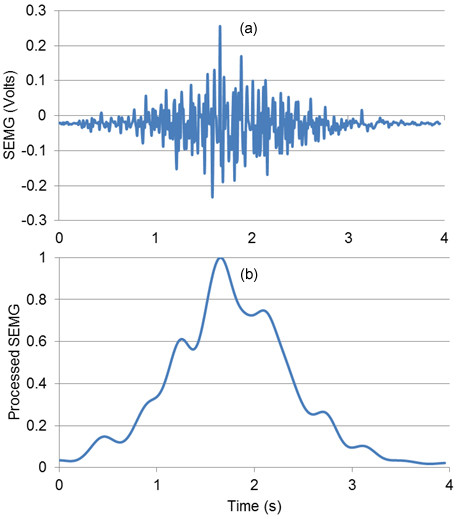
**Sample SEMG signal**. (a) Raw. (b) Filtered.

33,520 samples were acquired from each of the eight SEMG channels and the torque sensor for each volunteer. The data set was broken down into training and testing data. Figure 8 shows a sample of raw and processed SEMG signals.

## Results and Discussion

Models were initially trained with the training data set. The performance of trained models was subsequently tested by comparing estimated torque values from the model and the actual torque values from the testing data set. Two accuracy metrics were used to compare the performance of different models: normalized root mean squared error (NRMSE) and adjusted coefficient of determination $\left({\text{R}}_{\text{a}}^{2}\right)$[64]. Root mean squared error (RMSE) is a measure of the difference between measured and estimated values. NRMSE is a dimensionless metric expressed as RMSE over the range of measured torques values for each volunteer:

(13) $$\text{NRMSE = }\frac{\sqrt{\frac{\sum_{\text{i = 1}}^{\text{n}}{\left(\tau_{\text{e}}\left(\text{i}\right)-\tau_{\text{m}}\left(\text{i}\right)\right)}^{2}}{\text{n}}}}{\tau_{\text{m,flex}}+|\tau_{\text{m},\text{ext}}|}$$

where τ_e_(i) is the estimated and τ_m_(i) is the measured torque value for sample i, n corresponds to the total number of samples tested, and τ_m,flex _and τ_m,ext _are the maximum flexion and extension torques exerted by each volunteer. The absolute value of τ_m,ext _is considered because of the negative sign assigned to extension torque values during signal acquisition.

R^2 ^is a measure of the percentage of variation in the dependant variable (torque) collectively explained by the independent variables (SEMG signals):

(14) $${\text{R}}^{2}=1-\frac{\Sigma_{\text{i}=1}^{\text{n}}{\left(\tau_{\text{e}}\left(\text{i}\right)-\tau_{\text{m}}\left(\text{i}\right)\right)}^{2}}{\Sigma_{\text{i}=1}^{\text{n}}{\left(\tau_{\text{m}}\left(\text{i}\right)-\bar{\tau_{\text{m}}}\right)}^{2}}$$

where $\bar{\tau_{\text{m}}}$ is the mean measured torque.

However R^2 ^has a tendency to overestimate the regression as more independent variables are added to the model. For this reason, many researchers recommend adjusting R^2 ^for the number of independent variables:

(15) $${\text{R}}_{\text{a}}^{2}=1-\left[\left(\frac{\text{n}-1}{\text{n}-\text{k}-1}\right)\times \left(1-{\text{R}}^{2}\right)\right]$$

where ${\text{R}}_{\text{a}}^{2}$ is the adjusted R^2^, n is the number of samples and k is the number of SEMG channels.

Models were trained using every 100 data resampled from the processed signals to save model training time. Data set was reduced to 335 samples with resampling. Training time t, was measured as the number of seconds it took for each model to be trained. All training and testing was performed on a computer with an Intel^® ^Core™2 Duo 2.5 GHz processor and 6 GB of RAM. Table 3 compares mean training times for models trained using the original and resampled data sets.

**Table 3:** Model training times for original and resampled data sets.

Time (s)	PBM	OLS	RLS	SVM	ANN	LWPR
Original	1,080.07	0.01	1.98	19,125.31	166.73	5,195.03

Resampled	10.96	0.00	0.03	15.32	9.40	18.63

One-way Analysis of Variance (ANOVA) failed to reject the null hypothesis that NRMSE and ${\text{R}}_{\text{a}}^{2}$ have different mean values for each model, meaning that the difference between means is not significant (with minimum P-value of 0.95). We used reduced data sets with data resampled every 100 samples for the rest of the study.

### Number of Muscles

As merely one degree of freedom of the wrist was considered in this study, the possibility of training models using only two primary muscles was investigated initially. There are six combinations possible with one primary flexor and one primary extensor muscle: FCR-ECRL, FCR-ECRB, FCR-ECU, FCU-ECRL, FCU-ECRB, and FCU-ECU. Models were trained using 75% of the data for all six combinations and then tested on the remaining 25% and the model with the best performance was picked. Mean and standard deviation of NRMSE and ${\text{R}}_{\text{a}}^{2}$ for models trained with two, five, and eight channels are presented in Table 4.

**Table 4:** Comparison of joint torque estimation for models trained with two, five, and eight SEMG channels.

Model		8 channels		5 channels		2 channels	
		
		NRMSE	$R_{a}^{2}$	NRMSE	$R_{a}^{2}$	NRMSE	$R_{a}^{2}$
PBM	Mean	2.73%	0.85	3.07%	0.86	4.59%	0.77
	
	STD	0.97%	0.13	1.03%	0.11	1.32%	0.19

OLS	Mean	2.88%	0.84	3.17%	0.77	4.82%	0.63
	
	STD	0.94%	0.11	1.06%	0.13	1.81%	0.23

RLS	Mean	2.83%	0.82	3.11%	0.79	4.73%	0.69
	
	STD	0.93%	0.10	1.01	0.11	1.31%	0.18

SVM	Mean	2.85%	0.82	3.00%	0.80	4.77%	0.73
	
	STD	1.00%	0.09	1.04%	0.10	1.02%	0.14

ANN	Mean	2.82%	0.82	3.03%	0.81	4.74%	0.69
	
	STD	0.95%	0.09	1.05%	0.12	1.17%	0.18

LWPR	Mean	3.03%	0.75	3.19%	0.78	4.97%	0.69
	
	STD	1.14%	0.21	1.19%	0.13	1.31%	0.21

**Figure 9:**
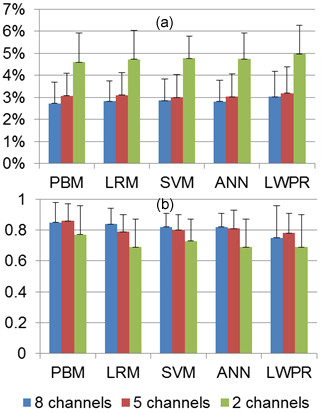
**Effects of the number of SEMG channels used for training on joint torque estimation**. (a) NRMSE. (b) ${\text{R}}_{\text{a}}^{2}$.

It is noteworthy that best performance was not consistently attributed to a single combination of muscles for the case of models trained with two channels. It is evident that models trained with five channels are superior to models trained with two. However models trained with eight channels do not have significant performance superiority. Figure 9 compares NRMSE and ${\text{R}}_{\text{a}}^{2}$ for different number of training channels.

This result appears to be in contrast to the results obtained by Delp et al. [66] where extrinsic muscles of the hand are expected to contribute substantially to torque generation. However, due to the design of our testing rig, volunteers only generated torque by pushing their palms against the torque-sensing plate and their fingers did not contribute to torque generation. Therefore the addition of SEMG signals of extrinsic muscles to the model did not result in a significant increase in accuracy.

It should be noted that using more data for training models increases accuracy for same session models. Table 5 compares NRMSE and ${\text{R}}_{\text{a}}^{2}$ for two extreme cases where 25% and 90% of the data set is used for training models and the rest of the data set is used for testing using all SEMG channels.

**Table 5:** Comparison of training data set size on joint torque estimation.

Model		25% training		90% training	
		
		NRMSE	$R_{a}^{2}$	NRMSE	$R_{a}^{2}$
PBM	Mean	4.41%	0.81	2.32%	0.96
	
	STD	2.49%	0.09	0.59%	0.04

OLS	Mean	4.19%	0.80	2.19%	0.97
	
	STD	2.19%	0.10	0.58%	0.04

RLS	Mean	4.14%	0.82	2.07%	0.97
	
	STD	2.13%	0.08	0.51%	0.03

SVM	Mean	4.39%	0.85	2.02%	0.97
	
	STD	2.46%	0.09	0.92%	0.03

ANN	Mean	5.87%	0.73	2.34%	0.96
	
	STD	2.20%	0.20	0.61%	0.03

LWPR	Mean	6.41%	0.69	3.43%	0.87
	
	STD	3.14%	0.29	0.84%	0.07

**Figure 10:**
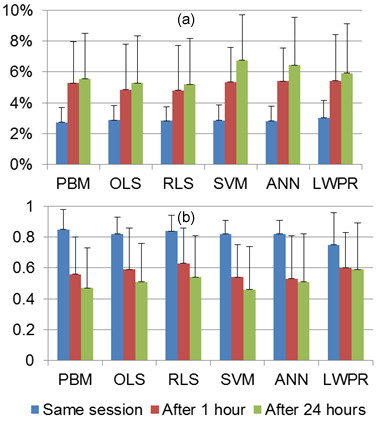
**Effects of training data size on joint torque estimation**. (a) NRMSE. (b) ${\text{R}}_{\text{a}}^{2}$.

Mean ${\text{R}}_{\text{a}}^{2}$ values increased 19%, 21%, 18%, 14%, 32%, and 26% while mean NRMSE values decreased 47%, 48%, 50%, 54%, 60%, and 46% for PBM, OLS, RLS, SVM, ANN, and LWPR, respectively. Figure 10 visualizes NRMSE and ${\text{R}}_{\text{a}}^{2}$ for the two cases.

For PBM training with two and five channels, ΣPCSA term in equation 7 was modified. For the two channel case, equation 7 took the following form:

(16) $$\begin{array}{c} \tau_{\text{e}}\left(\text{t}\right)=\frac{\Sigma \text{PCS}{\text{A}}_{\text{f}1\text{exors}}}{\text{PCS}{\text{A}}_{\text{f}1\text{exor}}}\times \tau_{\text{flexor}}\left(\text{t}\right)\\ +\frac{\Sigma \text{PCS}{\text{A}}_{\text{extensors}}}{\text{PCS}{\text{A}}_{\text{extensor}}}\times \tau_{\text{extensor}}\left(\text{t}\right) \end{array}$$

where ΣPCSA_flexors _is the summation of PCSA of all flexor muscles, ΣPCSA_extensors _is the summation of PCSA of all extensor muscles, PCSA_flexor _is the PCSA of the flexor muscle used for training, PCSA_extensor _is the PCSA of the extensor muscle used for training, τ_flexor_(t) is the torque of the flexor muscle used for training at time t, and τ_extensor_(t) is the torque of the flexor muscle used for training at time t.

Similarly PBM training with the five primary wrist muscles was carried out with modified ΣPCSA terms. Half of the summation of PCSA values for non-primary flexors was added to each of the two primary flexors while a third of the summation of PCSA values for non-primary extensors was added to the ΣPCSA term of each of the three primary extensors.

These modifications allowed tuned parameters to stay within their physiologically acceptable values, even though less SEMG channels were used for training models.

### Cross Session

Passages of time as well as electrode displacement adversely affect accuracy of models trained with SEMG [22,25]. Models trained with session 1 were tested with data from session 2 (in 1 hour without detaching electrodes) and session 3 (in 24 hours and with new electrodes attached). Table 6 compares model performance for the two cases.

**Table 6:** Effects of passage of time and electrode displacement on joint torque estimation.

Model		After 1 hour		After 24 hours	
		
		NRMSE	$R_{a}^{2}$	NRMSE	$R_{a}^{2}$
PBM	Mean	5.28%	0.56	5.54%	0.47
	
	STD	2.68%	0.24	2.95%	0.26

OLS	Mean	4.84%	0.59	5.29%	0.51
	
	STD	2.98%	0.27	3.04%	0.25

RLS	Mean	4.81%	0.63	5.19%	0.54
	
	STD	2.91%	0.23	2.98%	0.27

SVM	Mean	5.35%	0.54	6.76%	0.46
	
	STD	2.22%	0.21	2.95%	0.28

ANN	Mean	5.40%	0.53	6.44%	0.51
	
	STD	2.15%	0.28	3.09%	0.31

LWPR	Mean	5.42%	0.60	5.93%	0.59
	
	STD	3.00%	0.23	3.18%	0.30

**Figure 11:**
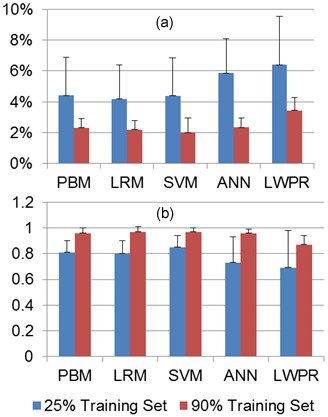
**Effects of passage of time and electrode displacement on joint torque estimation**. (a) NRMSE. (b) ${\text{R}}_{\text{a}}^{2}$.

Results suggest that model reliability deteriorates with passage of time. Figure 11 compares mean and standard deviation of NRMSE and ${\text{R}}_{\text{a}}^{2}$ of models trained with session 1 and tested with data from the same session, after 1 and 24 hours.

Mean ${\text{R}}_{\text{a}}^{2}$ values after one hour decreased 34%, 28%, 25%, 34%, 35%, and 20% while mean NRMSE decreased 93%, 68%, 70%, 88%, 91%, and 79% for PBM, OLS, RLS, SVM, ANN, and LWPR, respectively. After twenty four hours mean NRMSE values decreased further. High standard deviations of NRMSE and ${\text{R}}_{\text{a}}^{2}$ values suggest unreliability of model predictions with passage of time and electrode displacement. Therefore it is crucial for models trained using SEMG signals to be retrained frequently regardless of the model utilized.

### Arm Posture

Arm posture changes SEMG signal characteristics [8]. A model trained with the forearm in pronated position was utilized to predict the measured values from the supinated position in the same session. Supinating the forearm resulted in the torque sensor readings for extension and flexion to be reversed. This was explicitly taken into account when processing signals. Prediction accuracy of the trained models reduced significantly with forearm supination as shown in Table 7.

**Table 7:** Effects of forearm supination on joint torque estimation.

	Model	NRMSE	$R_{a}^{2}$
PBM	Mean	9.55%	0.22
	
	STD	5.69%	0.32

OLS	Mean	8.93%	0.25
	
	STD	5.37%	0.33

RLS	Mean	8.86%	0.23
	
	STD	5.30%	0.29

SVM	Mean	8.65%	0.24
	
	STD	4.47%	0.37

ANN	Mean	9.13%	0.23
	
	STD	4.76%	0.36

LWPR	Mean	10.05%	0.25
	
	STD	5.49%	0.30

**Figure 12:**
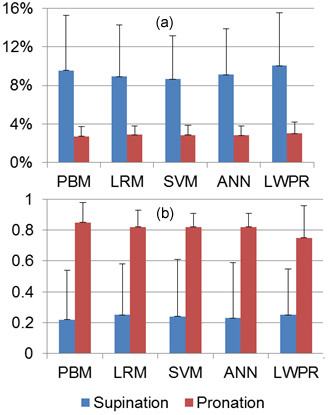
**Effects of arm posture on joint torque estimation**. (a) NRMSE. (b) ${\text{R}}_{\text{a}}^{2}$.

ANOVA shows that the hypothesis that NRMSE and ${\text{R}}_{\text{a}}^{2}$ of testing was the same is refuted with P < 0.01. Results from this experiment validate that trained models are very sensitive to arm posture. Forearm supination shifts SEMG signal space. Since models trained in the pronated position do not take this shift into consideration, accuracy decreases [22]. SEMG patterns change with different arm postures that models need to explicitly take into consideration [67,68]. Figure 12 shows the effects of forearm supination on prediction accuracy of models trained with forearm in pronated position. Mean NRMSE values increased 2.50, 2.10, 2.13, 2.04, 2.24, and 2.32 times for PBM, OLS, RLS, SVM, ANN, and LWPR.

Table 8 summarizes performance of models based on different criteria. One advantage of machine learning techniques is that these models can be trained with raw SEMG signals as they are capable of mapping the nonlinearities associated with raw SEMG signals. In contrast, PBM can only be trained with processed SEMG signals since inputs to the PBM represent neural activity of muscles (a value bounded between zero and one) [69]. Moreover, nonlinear behaviour of muscles [10] observed in raw SEMG signals precludes utilization of linear regression for mapping.

**Table 8:** Comparison of models investigated.

Criteria	PBM	OLS	RLS	SVM	ANN	LWPR
Least training time		*				

Physiological insight	*					

Does not require SEMG processing				*	*	*

Supination sensitivity	*	*	*	*	*	*

Time passage sensitivity	*	*	*	*	*	*

Electrode placement sensitivity	*	*	*	*	*	*

## Conclusions

Eleven volunteers participated in this study. During the first session, 33,520 samples from eight SEMG channels and a torque sensor were acquired while volunteers followed a protocol consisting of isometric flexion and extension of the wrist. We then processed SEMG signals and resampled every 100 samples to save model training time. Subsequently we trained models using identical training data sets. When using 90% of data as training data set and the rest of the data as testing data, we attained ${\text{R}}_{\text{a}}^{2}$ values of 0.96 ± 0.04, 0.97 ± 0.04, 0.97 ± 0.03, 0.97 ± 0.03, 0.96 ± 3, and 0.87 ± 0.07 for PBM, OLS, RLS, SVM, ANN, and LWPR respectively. All models performed in a very comparable fashion, except for LWPR that yielded lower ${\text{R}}_{\text{a}}^{2}$ values and higher NRMSE values.

Models trained using the data set from session one were tested using two separate data sets gathered one hour and twenty four hours following session one. We showed that Mean ${\text{R}}_{\text{a}}^{2}$ values after one hour decrease 34%, 28%, 25%, 34%, 35%, and 20% for PBM, OLS, RLS, SVM, ANN, and LWPR, respectively. Tests after twenty four hours showed even further performance deterioration. Therefore it was concluded that all models considered in this study are sensitive to passage of time and electrode displacement.

The effects of the number of SEMG channels used for training were explored. Models trained with SEMG channels from the five primary forearm muscles were shown to be of similar predictive ability compared to models trained with all eight SEMG channels. However, models trained with two SMEG channels resulted in predictions with lower ${\text{R}}_{\text{a}}^{2}$ and higher NRMSE values.

Finally models trained with forearm in a pronated position were tested with data gathered from forearm in the supinated position. Mean NRMSE values increased 2.50, 2.10, 2.13, 2.04, 2.24, and 2.32 times for PBM, OLS, RLS, SVM, ANN, and LWPR.

The substantial decrease in predictive ability of all models with passage of time, electrode displacement, and altering arm posture necessitates regular retraining of models in order to sustain estimation accuracy. We showed that resampling the data set substantially reduces the training time without sacrificing estimation accuracy of models. All models were trained in under 20 seconds while OLS was trained in under 10 ms. Low training times achieved in this work render regular retraining feasible.

## Competing interests

The authors declare that they have no competing interests.

## Authors' contributions

AZ designed the experiment, acquired SEMG data, carried out regression analysis, and drafted the manuscript. CM supervised the project, contributed to discussions and analysis and participated in manuscript revisions. All authors read and approved the final manuscript.
